# Experimental non-alcoholic steatohepatitis in Göttingen Minipigs: consequences of high fat-fructose-cholesterol diet and diabetes

**DOI:** 10.1186/s12967-019-1854-y

**Published:** 2019-04-03

**Authors:** Camilla Schumacher-Petersen, Berit Østergaard Christoffersen, Rikke Kaae Kirk, Trine Pagh Ludvigsen, Nora Elisabeth Zois, Henrik Duelund Pedersen, Mogens Vyberg, Lisbeth Høier Olsen

**Affiliations:** 1 Department of Veterinary and Animal Sciences, Faculty of Health and Medical Sciences, University of Copenhagen, Ridebanevej 9, 2., 1870 Frederiksberg, Denmark; 2 Global Drug Discovery, Novo Nordisk A/S, Novo Nordisk Park, 2760 Måløv, Denmark; 3 Department of Clinical Biochemistry, Copenhagen University Hospital Rigshospitalet, Blegdamsvej 9, 2100 Copenhagen Ø, Denmark; 4 In Vivo Pharmacology, Gubra ApS, Hørsholm Kongevej 11B, 2970 Hørsholm, Denmark; 5 Ellegaard Göttingen Minipigs A/S, Sorø Landevej 302, 4261 Dalmose, Denmark; 6 Institute of Pathology, Aalborg University Hospital, Ladegaardsgade 3, 9000 Aalborg, Denmark; 7 Department of Clinical Medicine, Aalborg University, Soendre Skovvej 15, 9000 Aalborg, Denmark

**Keywords:** NAFLD, NASH, Diabetes, Obesity, Metabolic syndrome, Dietary cholesterol, Animal model, Porcine

## Abstract

**Background:**

Non-alcoholic fatty liver disease (NAFLD) is the most common liver disease in humans, and ranges from steatosis to non-alcoholic steatohepatitis (NASH), the latter with risk of progression to cirrhosis. The Göttingen Minipig has been used in studies of obesity and diabetes, but liver changes have not been described. The aim of this study was to characterize hepatic changes in Göttingen Minipigs with or without diabetes, fed a diet high in fat, fructose, and cholesterol to see if liver alterations resemble features of human NAFLD/NASH.

**Methods:**

Fifty-four male castrated minipigs (age 6 to 7 months) were distributed into four groups and diet-fed for 13 months. Groups were: lean controls fed standard diet (SD, n = 8), a group fed high fat/fructose/cholesterol diet (FFC, n = 16), a group fed high fat/fructose/cholesterol diet but changed to standard diet after 7 months (diet normalization, FFC/SD, n = 16), and a streptozotocin-induced diabetic group fed high fat/fructose/cholesterol diet (FFC_DIA_, n = 14). At termination, blood samples for analyses of circulating biomarkers and liver tissue for histopathological assessment and analyses of lipids and glycogen content were collected.

**Results:**

In comparison with SD and FFC/SD, FFC and FFC_DIA_ pigs developed hepatomegaly with increased content of cholesterol, whereas no difference in triglyceride content was found. FFC and FFC_DIA_ groups had increased values of circulating total cholesterol and triglycerides and the hepatic circulating markers alkaline phosphatase and glutamate dehydrogenase. In the histopathological evaluation, fibrosis (mainly located periportally) and inflammation along with cytoplasmic alterations (characterized by hepatocytes with pale, granulated cytoplasm) were found in FFC and FFC_DIA_ groups compared to SD and FFC/SD. Interestingly, FFC/SD also had fibrosis, a feature not seen in SD. Only two FFC and three FFC_DIA_ pigs had > 5% steatosis, and no hepatocellular ballooning or Mallory–Denk bodies were found in any of the pigs.

**Conclusions:**

Fibrosis, inflammation and cytoplasmic alterations were characteristic features in the livers of FCC and FFC_DIA_ pigs. Overall, diabetes did not exacerbate the hepatic changes compared to FFC. The limited presence of the key human-relevant pathological hepatic findings of steatosis and hepatocellular ballooning and the variation in the model, limits its use in preclinical research without further optimisation.

## Background

It has recently been estimated that human non-alcoholic fatty liver disease (NAFLD) has a 25% worldwide prevalence and is expected to become the major reason for liver transplantations in the western world [1, 2].

NAFLD ranges from simple steatosis to non-alcoholic steatohepatitis (NASH), the latter characterized by additional inflammation, hepatocellular hydropic degeneration (also called ballooning) and eventually Mallory–Denk bodies associated with fibrosis which can lead to cirrhosis. Type 2 diabetes is considered the primary risk factor for progression of simple steatosis to advanced stages of NASH [3]. In addition, NAFLD seems to be a central risk factor for the development of cardiovascular disease [4] and other complications also related to diabetes [5, 6]. It can be difficult to diagnose NAFLD given that clinical signs usually are sparse even at late stages where liver damage is substantial; and changes in circulating liver biomarkers are often non-specific. Histopathological evaluation of samples from liver biopsies is still considered the gold standard for diagnosing the disease even though new non-invasive alternatives are emerging [7].

The hepatic disease mechanisms are still largely unknown, and effective treatment modalities are lacking. Therefore, various animal models have been investigated to help unravel the pathogenesis and for testing new pharmaceutical drug candidates or lifestyle interventions. Diet- or chemically-induced rodent models of NASH/NAFLD have mainly been used, and several mouse strains exist with spontaneous or transgenic mutations that pinpoint different signalling pathways [8, 9]. Larger animal models have also been examined, as anatomy, physiology or simply size can be beneficial depending on the research question of interest [10, 11].

Histopathological features resembling NASH in humans have been reported in Ossabaw minipigs [10, 12] fed a diet high in fat, fructose and cholesterol. Others have studied Bama minipigs and Microminipigs on high fat/high sucrose diet or high fat/high cholesterol diet with a supplement of cholic acid, respectively, and reported microvesicular steatosis and inflammation, but none or limited hepatocellular ballooning and fibrosis [11, 13]. The Göttingen Minipig is widely used in studies of obesity and metabolic syndrome [14–16], but diet-induced histopathological changes in the liver have not been characterized.

In this study, the aim was to investigate the liver changes in Göttingen Minipigs fed a diet with high content of fat, fructose and cholesterol for over a year. Hepatic gross morphology, histopathology and tissue content of lipids and glycogen as well as changes in relevant circulating biomarkers were investigated. Furthermore, it was evaluated if diabetes would exacerbate the alterations and if a diet change to standard diet would limit pathologic changes. The hypothesis was that Göttingen Minipigs fed a diet high in fat, fructose and cholesterol would develop hepatic changes that resemble human NAFLD including NASH.

## Materials and methods

### Study setup and animals

**Fig. 1:**
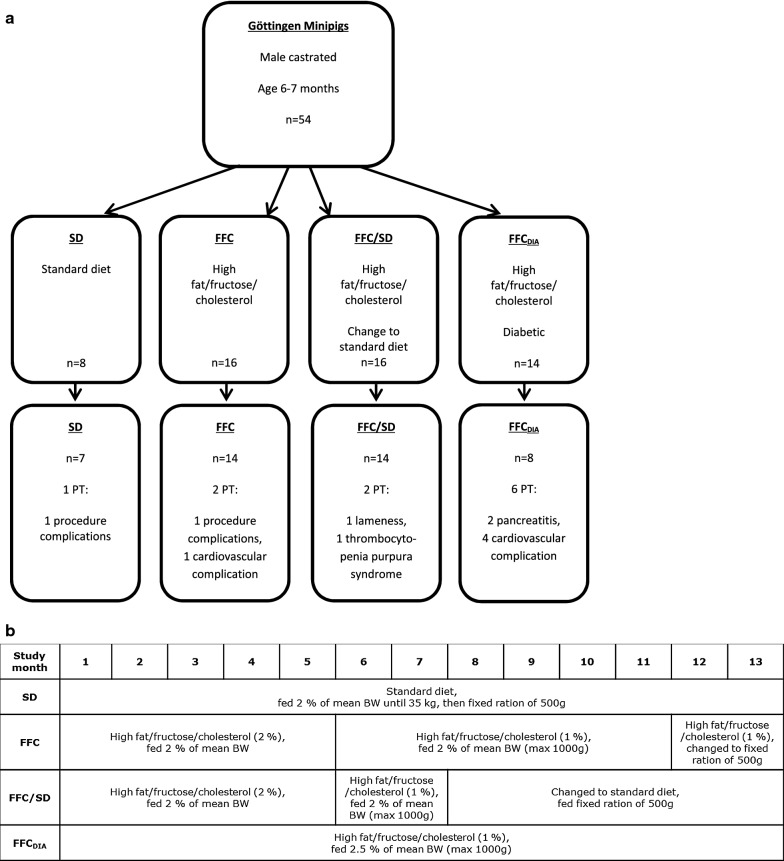
Flow diagram of the distribution of minipigs and details of diet feeding within each group. **a** Fifty-four Göttingen Minipigs were included in the study and eleven were terminated early. The reasons for early termination are provided under each group. The two cases of procedure-related complications occurred during investigations of other end points (unrelated to the present study) performed in the same animals. **b** Overview and details of the diet feeding in each of the four groups over the study period. *BW* body weight, *FFC* high fat/fructose/cholesterol diet group, *FFC*_*DIA*_ diabetic group, *FFC/SD* diet-normalization group, *PT* premature termination, *SD* lean control group

Castrated male Göttingen Minipigs (Ellegaard Göttingen Minipigs A/S, Dalmose, Denmark) (n = 54 in total) aged 6 to 7 months were weight stratified and distributed into four groups (Fig. 1a) and fed once daily for 13 months. The pigs were part of a larger study (n = 84 in total) looking at different aspects of obesity [17] and diabetes related complications (two intervention groups were not included in the present study). The included groups were: lean control pigs (SD, n = 8) fed standard diet (Mini-pig, SDS, UK (Table 1)); a group fed high fat/fructose/cholesterol (2%) diet (5B4L) for the first 5 months and subsequently changed to a similar diet with 1% cholesterol (9G4U) for the next 8 months [both diets from Test diet^®^, Missouri, USA (Table 1)] (high fat/fructose/cholesterol, FFC, n = 16); a group fed the same diets as FFC for the first 7 months but returned to standard diet the last 6 months of the study (diet-normalization, FFC/SD, n = 16) and a diabetic group fed a high fat/fructose/cholesterol (1%) diet throughout the study (9G4U) (FFC_DIA_, n = 14). Details of diet feeding in the four groups can be seen in Fig. 1b. Straw was used as bedding material, and the animals had access to fresh drinking water at all times.

**Table 1:** Nutritional composition of the three diets used in the study

Diet	Standard^a^	High fat, fructose, cholesterol (2%) (5B4L)^b^	High fat, fructose, cholesterol (1%) (9G4U)^b^
Carbohydrates (%)	74.6	40.8	40.8
Protein (%)	18.6	16.2	16.1
Fat (%)	6.8	43	43
Fructose (%)	5.5 (all sugars)	17.8	18.8
Cholesterol (ppm)	~ 0	20,045	10,045
Methionine (ppm)	1800	3500	3500
Choline (ppm)	784	668	668

Percentages are of total energy content

*ppm* parts per million

^a^Mini pig, Special Diet Services (SDS), Essex, United Kingdom

^b^TestDiets^®^, Missouri, USA

The diabetic group had type 1-like diabetes induced with streptozotocin administered intravenously once daily for three consecutive days [18] (modified version of Gerrity et al. optimized for Göttingen Minipigs; 60 mg/kg bodyweight). Diabetic animals were treated once daily with a long acting insulin (insulin glargine, Lantus^®^, Sanofi-Aventis Deutschland GmbH, Frankfurt am Main, Germany) subcutaneously immediately after the daily diet ration was offered in the morning; targeting fasting morning glucose levels of 14–16 mM.

Three to eight weeks before termination, blood for quantification of circulating biomarkers and intravenous glucose tolerance test (IVGTT) were sampled in plain serum tubes and EDTA coated tubes. Blood samples were kept on ice for maximum 30 min (EDTA) or kept at room temperature (serum) for 1 h before centrifugation for 10 min, 2000×*g* at 4 °C. After centrifugation EDTA plasma and serum were pipetted into appropriate tubes and stored at − 80 °C. The IVGTT procedure including calculation of intravenous glucose tolerance index (K_G_) and area under the curve for insulin response (AUC_Insulin_) were performed as previously described [17]. Homeostasis model assessment of insulin resistance (HOMA-IR) was calculated as fasting insulin (µU/mL) × fasting glucose (mM)/22.5 [19] in all animals except for the diabetic animals since their fasting glucose values were also influenced by the long-acting insulin glargine. Total body fat percentage (BF%) was estimated using dual energy x-ray absorptiometry (DEXA) (Lunar prodigy, GE Healthcare, Brøndby, Denmark) 1 to 6 weeks before termination. All animals were euthanized by exsanguination in general anaesthesia (mixture of zolazepam, tiletamine, ketamine, xylazine and butorphanol as previously described by Pedersen et al. [20]). After termination, the liver was removed and weighed. A sample (1 × 1 × 1–2 cm) from each of the four main liver lobes [*Lobus hepatis sinister medialis* (SM) and *lateralis* (SL), *Lobus hepatis dexter medialis* (DM) and *lateralis* (DL)] was collected mid-lobe and fixed in 10% neutral buffered formalin for 24–32 h, processed, and embedded in paraffin. From one liver lobe (SM) samples were obtained, snap frozen in liquid nitrogen and kept at − 80 °C or cryo fixated in optimum cutting temperature compound (OCT) (Tissue-Tek^®^ O.C.T. Compound, Sakura^®^ Finetek, Alphen aan den Rijn, The Netherlands) on dry ice. Animals were fasted overnight before blood sampling, DEXA assessment, and termination.

### Analysis of circulating biomarkers

Plasma concentrations of alkaline phosphatase (ALP), alanine transaminase (ALT), aspartate transaminase (AST), glutamate dehydrogenase (GLDH), total cholesterol (TC), triglycerides (TG), glucose (GLU), fructosamine (FRA), and albumin (ALB) were quantified using an autoanalyzer Cobas 6000^®^ (Roche A/S, Hvidovre, Denmark). A modified version of a previously described method [21] was used for quantification of C-reactive protein in serum (CRP).

### Biochemical analysis of liver tissue content of triglycerides, cholesterol and glycogen

Snap frozen SM liver samples were homogenized as previously described [22] and the content of cholesterol, triglycerides and glycogen were measured by Cobas 6000^®^ in duplicates. Six samples were analyzed six times for the determination of intra-assay variation.

### Histopathological assessment

The liver samples were fixed in neutral buffered formalin for 24–32 h. From each lobe one six mm tissue core was punched. The cores were embedded in paraffin in a recognisable pattern [tissue micro array (TMA)]. Three µm thick sections were cut and routinely stained with hematoxylin and eosin (HE), picro-sirius red (PSR), and periodic acid-Schiff (PAS) with and without diastase pre-treatment. Selected elements from the NAFLD activity score (NAS) [23] were used to evaluate steatosis (HE), fibrosis (PSR) and inflammation (HE). The latter was evaluated by the number of inflammatory foci (defined as at least 5 extravascular inflammatory cells in a cluster). Cytoplasmic alterations (CA) in the hepatocytes (HE) and the glycogen content (PAS) were scored from 0 (none) to 3 (severe). For all findings, the localization or zonal distribution was recorded. In Table 2, an overview of the different elements in the qualitative histopathological assessment is provided. All assessments were performed in a blinded manner by one observer and, moreover, eight samples were scored three times for the determination of intra-observer variation.

**Table 2:** Overview of elements in the qualitative histopathological assessment

Elements from human diagnostics	Score	Type/localization
Steatosis (NAS)	0: < 5% 1: 5–33% 2: > 33–66% 3: > 66%	Macrovesicular, microvesicular or mixed Zone 1, 2, 3
Fibrosis (NAS)	0: None 1: Perisinusoidal or periportal 2: Perisinusoidal and portal/periportal 3: Bridging 4: Cirrhosis	Subgroups of score 1: 1A: Mild, zone 3, perisinusoidal 1B: Moderate, zone 3, perisinusoidal 1C: Portal/periportal
Inflammatory foci per 200× field, mean of 3 fields (NAS)	0: 0 1: < 2 2: 2–4 3: > 4	Lobular or portal
Hepatocellular ballooning	0: None 1: Few balloon cells 2: Many cells/prominent ballooning	Hydropic degeneration
Mallory–Denk bodies	0: None to rare 1: Many	

Other elements	Score	Localization
Cytoplasmic alterations	0: − 1: + 2: ++ 3: +++	Zone 1, 2, 3
PAS positive staining	0: − 1: + 2: ++ 3: +++	Zone 1, 2, 3

For each element scoring, localization and/or type were recorded

*NAS* Modified from the NAFLD activity score (NAS) [22], *PAS* Periodic acid Schiff (glycogen staining)

Immunohistochemical (IHC) staining using a polyclonal goat anti-ionized calcium binding adapter molecule 1 (Iba1) antibody (Abcam #ab5076) was applied to detect macrophages [24]. Antigen retrieval on deparaffinized slides was done by microwave heating in TEG-buffer pH 9.0 (Ampliqon A/S, Odense, Denmark) for 15 min before endogenous peroxidase was inhibited with hydrogen peroxide (3%) (Merck, Darmstadt, Germany) for 10 min. Both avidin and biotin (Dako Biotin blocking system, Dako A/S, Glostrup, Denmark) was used for 10 min each for blockage of endogenous biotin. Pre-incubation with donkey serum (7%) (Jackson Immunoresearch, West Grove PA, USA) in Tris-buffered saline (TBS) (Ampliqon A/S, Odense, Denmark) + Tween 20 (TBS + T) (Merck, Darmstadt, Germany) and skimmed milk (5%) (BD, Kgs. Lyngby, Denmark) for blockage of unspecific antibody binding was performed for 30 min. Primary goat antibody against Iba1 (1:1500) (Abcam, Cambridge, UK, #5076) was applied for 120 min; thereafter secondary antibody (biotinylated donkey-anti-goat 1:1000) (Jackson Immunoresearch, West Grove PA, USA, #705-065-147) was applied for 30 min. Both antibodies were dissolved in a TBS + T + 0.5% skimmed milk solution. ABC-detection system (VECTASTAIN^®^ Elite^®^ ABC-HRP Kit, Vector Laboratories, Burlingame, CA, USA) was used for 30 min followed by chromogen (DAB + chromogen, Dako A/S, Glostrup, Denmark) for 10 min before counterstaining was performed with Mayer’s hematoxylin (Sigma-Aldrich/Merck, Darmstadt, Germany). Between every step, except blockage of unspecific antibody binding, slides were washed with TBS + T. A negative control slide was used without primary antibody, and two different non-conjugated goat IgG were used for check of unspecific staining [‘ChromPure Goat IgG’ (1:10,000), Jackson Immunoresearch, West Grove PA, USA, #005-000-003 and ‘Normal Goat IgG Control’ (1:1000), R&D Systems, Oxon, UK, #AB-108-C].

Also, 10 µm sections from OCT embedded frozen blocks from lobe SM were cut and stained with Oil red O (ORO) in order to visualize the distribution of lipid and confirm steatosis found on HE sections. Image analysis software (Visiopharm, Hørsholm, Denmark) was used to quantify fibrosis (PSR-VIS), inflammation (Iba1-VIS) and lipid (ORO-VIS) by measuring the stained areas. For all TMAs (PSR-VIS and Iba1-VIS), the measured area was adjusted for the number of lobules in order to normalise for the difference in lobular diameter. The lobular diameter was estimated as the number of all lobules represented (both whole and fractioned) per TMA, since all areas of TMAs were nearly the same; a low number indicating a larger lobular diameter. Total tissue area for frozen OCT samples (ORO-VIS) varied in size which led to correction of the stained area with total tissue area.

### Statistical analysis

Shapiro–Wilk test was used to test whether data were normal distributed in groups and due to the majority of the evaluated parameters not being normally distributed, all data were expressed in tables as medians and interquartiles.

Differences among the four liver lobes in histopathology were investigated by a linear model taking repeated measurements into account.

Group differences in basic characteristics, circulating biomarkers, liver tissue content, and histopathological parametric data were tested using analysis of variance (ANOVA) with Welch adjustment for unequal variance between groups and Tukey’s correction for multiple comparisons. When needed, an appropriate transformation of the response variables was performed to obtain variance homogeneity and normal distribution of residuals in the ANOVA analyses. Fisher’s exact test was used to test categorical data for group differences.

In addition, linear regression was used to test for associations between histopathological data and circulating biomarkers or tissue content. Associations between histopathological scores were evaluated using Fisher’s exact test.

Statistical analyses were performed using SAS 9.4 software (SAS Institute Inc., Cary, NC, USA) and the significance level was set to *p *< 0.05. Graphs were created with GraphPad Prism (GraphPad Software Inc., La Jolla, CA, USA).

## Results

Forty-three pigs completed the study. Eleven pigs were excluded (Fig. 1a). Five pigs were euthanized according to predefined endpoints and six died suddenly: one SD (euthanized due to procedure related complications), two FFC (one euthanized due to procedure related complications and another died suddenly with a tentative diagnosis of circulatory failure with accumulation of serosanguinous fluid or blood in body cavities), two FFC/SD [one due to lameness, another due to spontaneous acute bleeding (diagnosed thrombocytopenia purpura)] and six FFC_DIA_ (two due to severe necrosis/inflammation of pancreas), where one died suddenly, and four died suddenly due to pulmonary embolus (n = 3), or a tentative diagnosis of circulatory failure with accumulation of serosanguinous fluid or blood in body cavities (n = 1). Diagnoses were based on clinical signs and gross pathology.

**Fig. 2:**
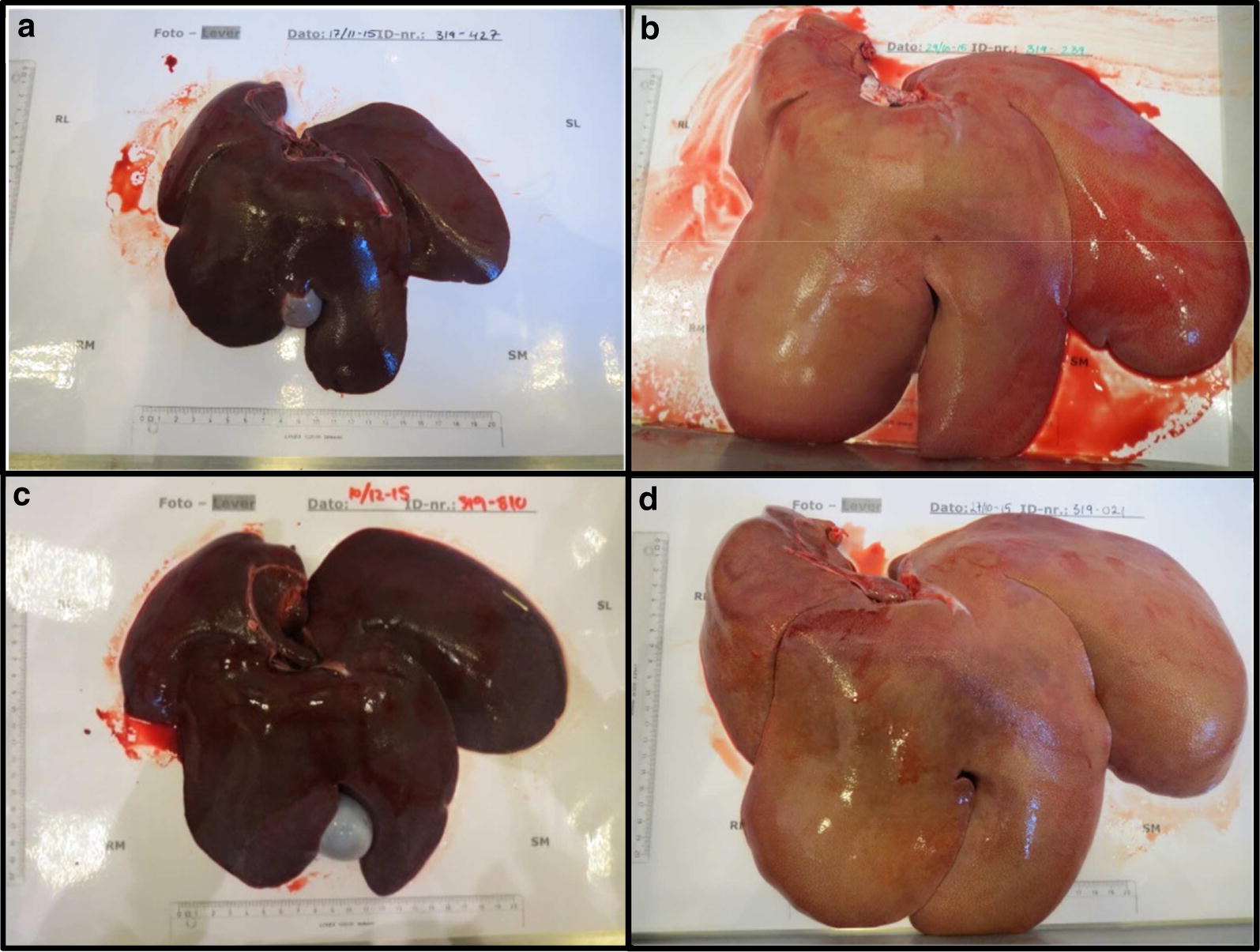
Liver gross morphology. Images show the typical liver gross morphology in each diet group. Bars (partly) visible on the sheet to the left and below the livers represent 20 cm. **a** *SD* Lean control group. **b** *FFC* high fat/fructose/cholesterol diet group. **c** *FFC/SD* diet-normalization group. **d** *FFC*_*DIA*_ diabetic group

Higher body weight (BW), liver weight (LW) and total body fat percentages (BF%) were found in FFC and FFC_DIA_ groups compared to the SD group, whereas only BW and BF% were higher in the FFC/SD group (Table 3). By macroscopic inspection, the majority of livers from FFC and FFC_DIA_ pigs were enlarged, pale red or yellow in color and with a bulgy appearance compared to SD. Livers from FFC/SD animals did not differ markedly from SD in size, color and gross appearance (Fig. 2).

**Table 3:** Basic characteristics and circulating biomarkers of the four study groups

Diet group	SD n = 7	FFC n = 14	FFC/SD n = 14	FFC_DIA_ n = 8	Over all *p*-value
Basic characteristics					
BW (kg)	39.00 (38.00; 41.00)	78.00 (69.00; 81.00)^a^	54.50 (51.00; 59.00)^ab^	59.50 (54.25; 64.00)^ab^	< 0.0001^^^
LW (g)	485 (458; 564)	1732 (1067; 2219)^a^	703 (627; 772)^b^	2077 (1478; 2439)^a^	< 0.0001^^^
LW:BW	0.013 (0.011; 0.015)	0.021 (0.014; 0.031)^a^	0.013 (0.012; 0.014)^b^	0.036 (0.028; 0.043)^ab^	< 0.0001^^^
BF% (%)	27.60 (24.00; 30.70)	64.20 (61.40; 67.60)^a^	47.30 (41.20; 50.20)^ab^	54.80 (52.65; 56.00)^ab^	< 0.0001
Basic circulating biomarkers					
TC^1^ (mmol/L)	1.70 (1.64; 2.18)	11.94 (11.00; 13.18)^a^	1.89 (1.56; 2.00)^b^	18.91 (16.91; 27.00)^ab^	< 0.0001^^^
TG^1^ (mmol/L)	0.34 (0.29; 0.35)	0.63 (0.54; 0.88)^a^	0.36 (0.32; 0.45)^b^	1.45 (0.57; 1.72)^ab^	0.0002^^^
GLU^1^ (mmol/L)	3.48 (3.32; 3.67)	3.72 (3.60; 3.83)	3.73 (3.47; 3.95)	15.1 (14.67; 15.45)^ab^	< 0.0001^^^
FRA^1^ (µmol/L)	247 (245; 271)	240 (235; 254)	246 (242; 251)	535 (452; 566)^ab^	< 0.0001^^^
K_G_ (min^−1^)^2^	3.15 (2.74; 3.46)	2.11 (1.82; 2.38)^a^	3.2 (2.5; 3.6)^b^	0.74 (0.62; 0.81)^a,b^	< 0.0001^^^
AUC_Insulin_ (pM * min)^3^	12,573 (11,507; 20,683)	27,168 (17,204; 35,562)	24,032 (20,478; 28,147)	800 (616–1310)^a,b^	< 0.0001^^^
HOMA-IR^4^	0.47 (0.35; 0.42)	1.11 (0.84; 1.58)^a^	1.59 (1.00; 1.93)^a^	NA	0.005^^^
ALB^1^ (g/L)	45.10 (42.70; 46.40)	42.20 (38.70; 46.20)	45.60 (42.80; 47.55)	45.45 (32.30; 46.00)	0.6
CRP^1^ (ng/ml)	2320 (1840; 14,100)	3160 (2760; 6780)	4510 (2890; 7130)	7820 (3260; 19,600)	0.7^^^
Hepatic circulating biomarkers					
ALP^1^ (U/L)	62.5 (57.0; 67.0)	209.0 (143.0; 366.0)^a^	76.0 (61.5; 91.0)^b^	177.5 (139.0; 542.0)^a^	< 0.0001^^^
ALT^1^ (U/L)	61.00 (51.60; 67.70)	34.50 (27.20; 48.40)^a^	52.25 (40.75; 60.60)^b^	47.60 (34.40; 68.50)	0.02^^^
AST^1^ (U/L)	33.90 (23.30; 96.10)	63.60 (25.50; 98.40)	49.65 (32.65; 59.75)	89.10 (45.40; 103.20)	0.2^^^
AST:ALT^1^	0.64 (0.49; 1.19)	1.51 (0.85; 2.03)^a^	0.80 (0.70; 1.07)	1.73 (1.23; 2.3)^a^	0.02^^^
GLDH^1^ (U/L)	2.30 (1.80; 3.20)	6.1 (3.20; 17.80)^a^	4.70 (3.35; 5.65)	15.80 (14.50; 23.40)^a^	0.0003^^^

Results are presented as median and 25% and 75% quartiles. Results for BW, BF%, TC, TG, KG and AUC_Insulin_ in diet group SD, FFC and FFC/SD have been presented previously [17]

*ALB* albumin, *ALP* alkaline phosphatase, *ALT* alanine transaminase, *AST* aspartate transaminase, AUC_Insulin_ area under the curve of insulin, *BF%* total body fat percentage, *BW* body weight, *CRP* C-reactive protein, *FRA* fructosamine, *GLDH* glutamate dehydrogenase, *GLU* glucose, *FFC* high fat/fructose/cholesterol diet group, *FFC*_*DIA*_ diabetic group, *FFC/SD* diet-normalization group, HOMA-IR homeostasis model assessment of insulin resistance, K_G_ intravenous glucose tolerance index, *LW* liver weight, *LW:BW* liver weight:body weight, NA not applicable, SD lean control group, *TC* total cholesterol, *TG* triglycerides

^^^Transformed

^a^Significantly different from SD

^b^Significantly different from FFC

^1^n = 6 for SD, n = 13 for FFC, n = 12 for FFC/SD, n = 6 for FFC_DIA_, due to catheter failure

^2^n = 6 for SD, n = 13 for FFC, n = 9 for FFC/SD, n = 6 for FFC_DIA_

^3^n = 5 for SD, n = 13 for FFC, n = 8 for FFC/SD, n = 4 for FFC_DIA_

^4^n = 6 for SD, n = 14 for FFC, n = 10 for FFC/SD

### Circulating biomarkers

Circulating hepatic biomarkers ALP and GLDH were increased in FFC and FFC_DIA_ groups compared to SD, whereas the FFC group had lower values of ALT (Table 3). No statistically significant difference was found for AST or the inflammation markers CRP and ALB. FFC and FFC_DIA_ groups had higher TG and TC than SD and FFC/SD groups with TC further elevated in FFC_DIA_ compared to FFC. As expected, fasting GLU and FRA values were significantly elevated in the FFC_DIA_ group compared to the three other groups, and in addition the FFC_DIA_ group had significantly lower K_G_ and AUC_Insulin_ than the other groups (Table 3). Furthermore, K_G_ in FFC was significantly decreased compared to SD and FFC/SD, suggesting impaired glucose metabolism in the two groups FFC and FFC_DIA_. The lower K_G_ despite a numerically ~ twofold higher AUC_insulin_ in the FFC group compared to SD indicates that the FFC diet and following obesity induces insulin resistance, which is also supported by the significantly higher HOMA-IR in this group compared to SD. The additional lowering of K_G_ in the FFC_DIA_ group compared to the FFC group is due to the insulin depletion conferred by the STZ-procedure. The only difference between FFC/SD and SD was a significantly higher HOMA-IR in the FFC/SD, indicating that with this index insulin resistance had not completely normalised yet.

### Biochemical analyses of liver tissue content

**Fig. 3:**
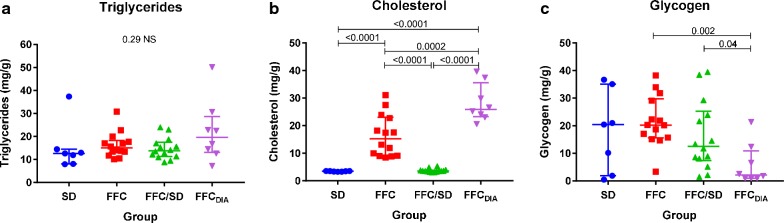
Group difference in liver tissue content of lipids, cholesterol, and glycogen. Biochemical liver tissue content of **a** triglycerides, **b** cholesterol and **c** glycogen for each diet group. Bars represent median and interquartile intervals. *p*-values from log transformed outcome, *NS* non-significant. *FFC* high fat/fructose/cholesterol diet group, *FFC*_*DIA*_ diabetic group, *FFC/SD* diet-normalization group, *SD* lean control group

Liver tissue content of triglyceride content did not differ between the groups (Fig. 3a), but cholesterol was higher in FFC and FFC_DIA_ groups compared to SD and FFC/SD groups (Fig. 3b). Regarding glycogen, FFC_DIA_ had lower liver content than FFC (Fig. 3c).

### Histopathological changes

**Fig. 4:**
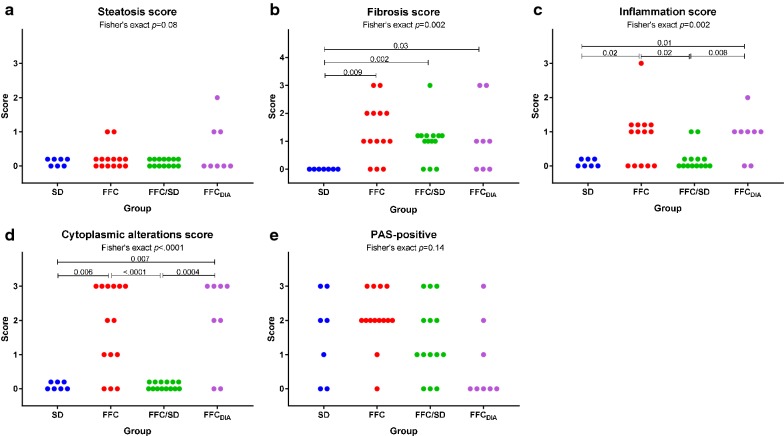
Difference between groups for histopathological scores. Scores for **a** steatosis, **b** fibrosis, **c** inflammation, **d** cytoplasmic alterations and **e** PAS-positive staining for each diet group. *FFC* high fat/fructose/cholesterol diet group, *FFC*_*DIA*_ diabetic group, *FFC/SD* diet-normalization group, *SD* Lean control group

Only few and minor differences in histopathological changes were observed among the four liver lobes indicating that the alterations were evenly distributed in the liver. Only slightly higher steatosis scores were found in SL (4 out of 43 pigs had score ≥ 1) and SM (5/43) compared to DL and DM (both 0/43) (*p *< 0.0001), and regarding inflammatory foci, DM (20/43) scored higher than DL (13/43) (*p *= 0.03). Further detailed evaluation of the liver lobe SM showed that steatosis, predominantly of macrovesicular type, was found in pigs from FFC and FFC_DIA_ groups with a centrilobular distribution, but only five animals (FFC = 2, FFC_DIA_ = 3) had ≥ 5% parenchymal involvement and no significant difference was found among groups (Fig. 4).

An increased content of collagen was found in FFC, FFC_DIA_ and FFC/SD groups in comparison to SD. The majority of these pigs had periportal fibrosis; however, four pigs (all FFC) had both periportal and randomly distributed lobular fibrosis. In addition, five pigs (FFC = 2, FFC/SD = 1, FFC_DIA_ = 2) showed porto-central bridging fibrosis (Fig. 4). None of the cases showed centrilobular pericellular fibrosis.

The number of lobular inflammatory foci was increased in FFC and FFC_DIA_ groups compared to both SD and FFC/SD (Fig. 4).

**Fig. 5:**
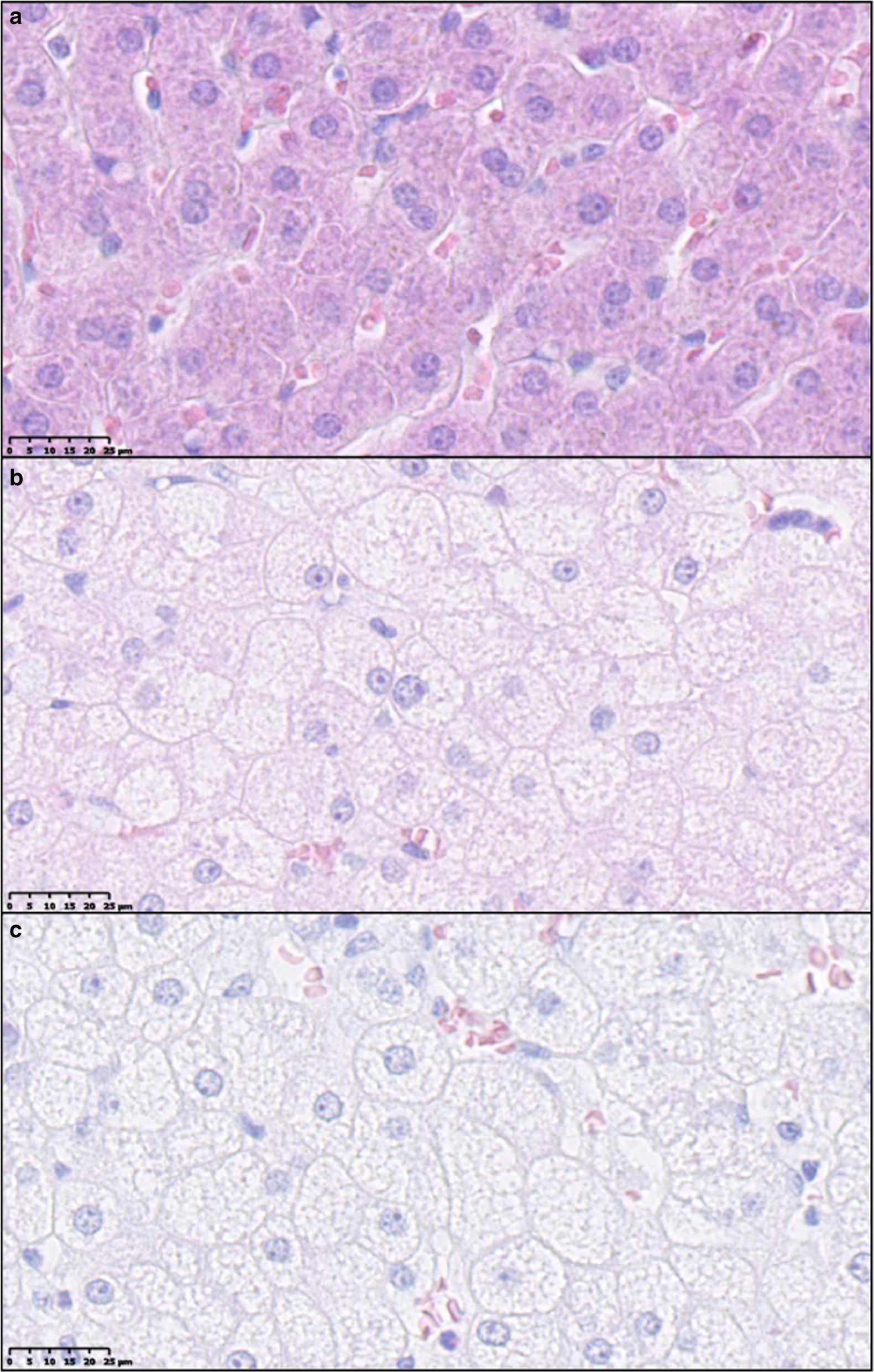
Examples of cytoplasmic alterations in hepatocytes characterized by hepatocytes with pale, granular appearance. **a** Normal hepatocytes from lean control animal (SD). **b**, **c** Hepatocytes with cytoplasmic alterations both from animals fed high fat/fructose/cholesterol diet (FFC). Scale bar 25 µm. Hematoxylin and eosin staining

Hepatocellular cytoplasmic alterations, characterized by hepatocytes with pale granular cytoplasm (Fig. 5), were a characteristic feature in most FFC and FFC_DIA_ pigs, in contrast to both SD and FFC/SD, where no alterations were seen (Fig. 4). These changes were homogenous and widely distributed and appear to be similar to those seen in Ossabaw minipigs using the same diet [10, 12]. The changes have some resemblance to the hepatocellular ballooning in human NASH such as rounded hepatocyte enlargement and presence of pale cytoplasm but lack key characteristics such as cytoplasmatic vacuoles and Mallory–Denk bodies [25]. Further, no difference among groups was found for glycogen staining with PAS with or without pre-treatment with diastasis (Fig. 4); in contrast to the biochemical analysis that showed FFC_DIA_ had less liver tissue glycogen content than FFC.

**Fig. 6:**
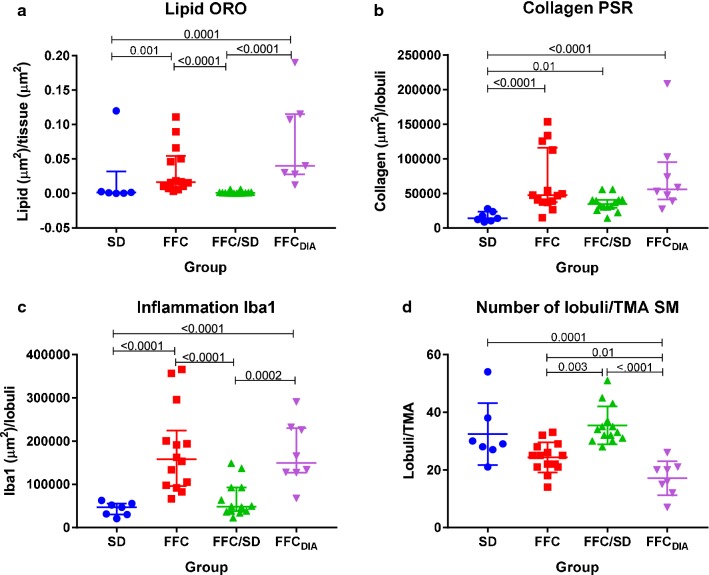
Parametric histopathological assessment of lipid, collagen and inflammation plus difference in lobules/TMA between groups. Differences between diet groups. **a** Quantification of lipid on Oil red O staining. **b** Quantification of collagen as a measure for fibrosis on Picro-sirius red staining. **c** Quantification of anti-Iba1 immuno-positive macrophages as a measure of inflammation. **d** Difference between groups in the number of lobules per TMA from the lobe SM. Bars represent median and interquartile intervals. *p*-values are from log transformed outcome. *FFC* high fat/fructose/cholesterol diet group, *FFC*_*DIA*_ diabetic group, *FFC/SD* diet-normalization group, *SD* Lean control group, *SM* lobus hepatis sinister medialis, *TMA* tissue micro arrays

**Fig. 7:**
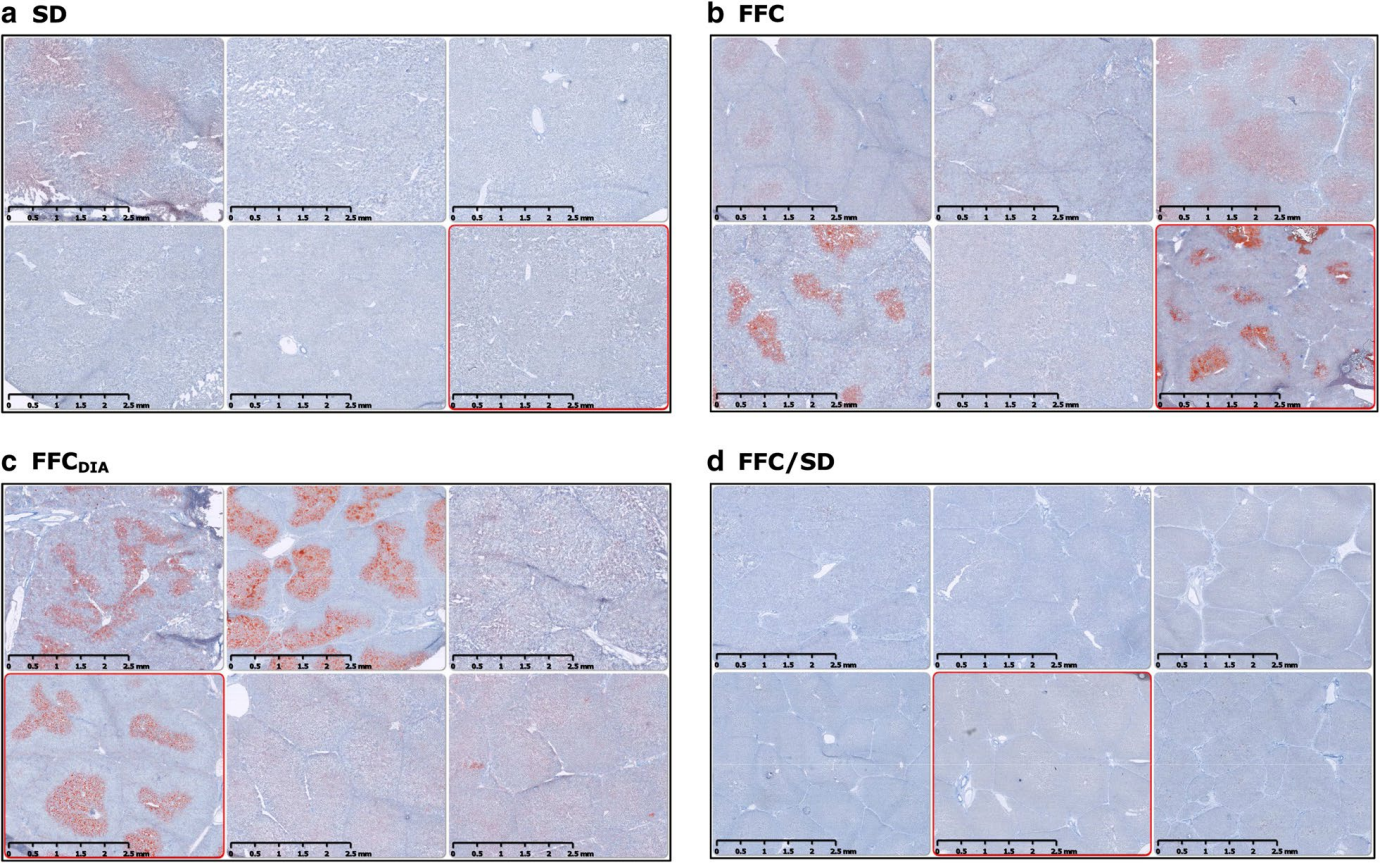
Heterogeneity of Oil red O staining of lipid within the diet groups. Six representative examples of lipid staining from six different animals within each group showing different patterns of lipid distribution. Scale bar 2.5 mm. **a** *SD* Lean control group. **b** *FFC* high fat/fructose/cholesterol diet group. **c** *FFC*_*DIA*_ diabetic group. **d** *FFC/SD* diet-normalization group

**Fig. 8:**
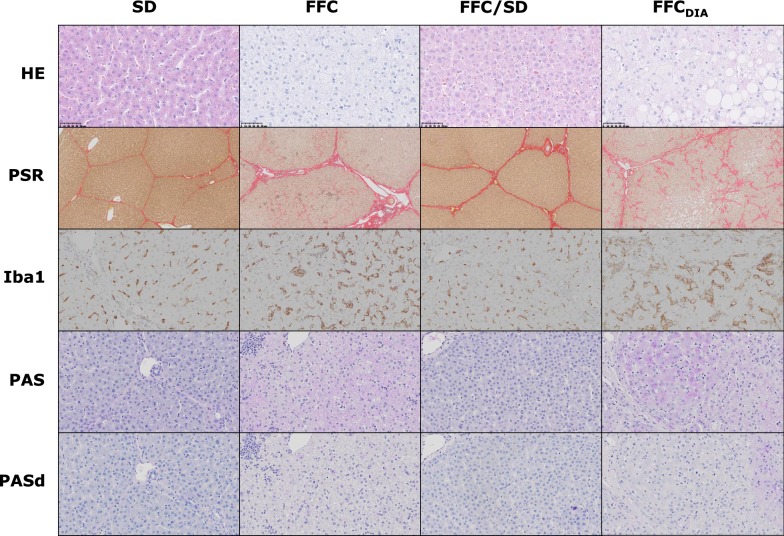
Panel of representative histopathological findings in the different diet groups. 1st row: SD + FFC/SD normal hepatocytes without cytoplasmic alterations (CA) (CA score 0). FFC Hepatocytes with cytoplasmic alterations (CA score 3). FFC_DIA_ Hepatocytes with cytoplasmic alterations (CA score 3) plus macrovesicular steatosis (steatosis score 1). Hematoxylin and eosin (HE) staining, magnification ×40. 2nd row: SD Normal collagen septa surrounding the lobules (fibrosis score 0). FFC Periportal and perisinusoidal fibrosis (fibrosis score 2). FFC/SD Portal/periportal fibrosis seen as excessive collagen deposition along septa and portal areas (fibrosis score 1C). FFC_DIA_ Bridging fibrosis with collagen deposition from portal area to central vein (fibrosis score 3). Picro-sirius red (PSR) staining, magnification ×5. 3rd row: SD + FFC/SD normal background staining of macrophages. FFC + FFC_DIA_ Increased staining of macrophages compared to normal. Immunochemical staining with antibodies against Iba1, magnification ×20. 4th row: Periodic-acid Schiff (PAS) staining for glycogen SD ‘+’ (PAS positive score 1). FFC ‘++’ (PAS positive score 2). FFC/SD ‘−’ (PAS positive score 0). FFC_DIA_ ‘++’ (PAS positive score 2). PAS staining without pretreatment with diastasis, magnification ×20. 5th row: SD + FFC + FFC/SD + FFC_DIA_ Pretreatment with diastasis for degradation of glycogen before staining with periodic-acid Schiff (PASd). Used as control for intensity of glycogen (PAS) staining without pretreatment, magnification ×20. *SD* Lean control group, *FFC* high fat/fructose/cholesterol diet group, *FFC/SD* diet-normalization group, *FFC*_*DIA*_ diabetic group

For all TMAs, total tissue area was nearly the same whereas the number of lobules within that area varied between pigs from seven to fifty-four (lower number = larger lobular diameter). The FFC_DIA_ group had larger lobular diameter than the three other groups (all *p *< 0.01), while the lobular diameter in the FFC group was also larger than in the FFC/SD group (*p *= 0.003) (Fig. 6). Quantification of collagen content using PSR revealed excessive collagen for FFC, FFC_DIA_ and FFC/SD compared to SD (Fig. 6). When staining with ORO, larger areas of lipids were found for FFC and FFC_DIA_ groups compared to both SD and FFC/SD groups (Fig. 6); however, a large degree of heterogeneity was seen within the groups (Fig. 7). IHC staining for Iba1 indicated increased number of macrophages in both FFC and FFC_DIA_ groups as compared to SD and FFC/SD groups (Fig. 6). Figure 8 shows typical examples of histopathological findings for each group.

### Associations between circulating biomarkers and histopathology

Regarding associations between circulating biomarkers and parametric histopathological variables (Table 4), lipid parameters TC and TG were associated with both ORO-VIS and Iba1-VIS, whereas GLDH was associated with ORO-VIS and ALP to Iba1-VIS.

**Table 4:** Associations between the circulating biomarkers and the histopathological findings

Elements	ORO-VIS	PSR-VIS	Iba1-VIS	Steatosis score	Fibrosis score	Inflammation score	CA score	PAS score
Biomarkers								
TC	< 0.0001 (0.41)^a^	0.01 (0.17)^a^	< 0.0001 (0.45)^a^	0.05 (0.16)^a^	0.2 (0.14)	0.02 (0.26)^a^	< 0.0001 (0.56)^a^	0.3 (0.10)
TG	0.001 (0.28)^a^	0.01 (0.17)^a^	0.0007 (0.28)^a^	0.2 (0.10)	0.04 (0.22)^a^	0.009 (0.29)^a^	< 0.0001 (0.49)^a^	0.4 (0.09)
ALP	0.003 (0.23)^a^	0.002 (0.25)^a^	0.0006 (0.29)^a^	0.11 (0.12)	0.04 (0.22)^a^	0.008 (0.30)^a^	< 0.0001 (0.55)^a^	0.03 (0.24)^a^
ALT	0.2 (0.06)	0.4 (0.02)	0.005 (0.20)^a^	0.02 (0.21)^a^	0.6 (0.05)	0.5 (0.07)	0.1 (0.15)	0.1 (0.15)
AST	0.02 (0.16)^a^	0.01 (0.17)^a^	0.2 (0.05)	0.1 (0.13)	0.01 (0.27)^a^	0.2 (0.13)	0.03 (0.23)^a^	0.9 (0.02)
AST:ALT	0.0005 (0.31)^a^	0.0001 (0.35)^a^	0.006 (0.20)^a^	0.02 (0.20)^a^	< 0.0001 (0.47)^a^	0.008 (0.30)^a^	0.0004 (0.42)^a^	0.5 (0.07)
GLDH	0.001 (0.27)^a^	0.007 (0.19)^a^	0.003 (0.22)^a^	0.2 (0.10)	0.002 (0.35)^a^	0.03 (0.23)^a^	0.0002 (0.44)^a^	0.7 (0.05)
CRP	0.7 (0.01)	0.6 (0.01)	0.2 (0.04)	0.5 (0.03)	0.8 (0.03)	0.9 (0.02)	0.7 (0.04)	1 (0.00)

All response variables were transformed for normal distribution of residuals

*ALP* alkaline phosphatase, *ALT* alanine transaminase, *AST* aspartate transaminase, *CRP* C-reactive protein, *CA* cytoplasmic alterations, *GLDH* glutamate dehydrogenase, *Iba1*-*VIS* quantification of macrophages on Iba1 immunohistochemical stain, *ORO*-*VIS* quantification of lipid on oil red O stain, *PAS* positive periodic acid Schiff stain for glycogen, *PSR*-*VIS* quantification of collagen on picro-sirius red stain, *TC* total cholesterol, *TG* triglycerides

Data presented as *p*-value (R^2^)

^a^Significant result

Comparison between circulating biomarkers and categorical histopathological features (Table 4), revealed association between both circulating lipids TG and TC and the inflammation score and especially the CA score. For circulating hepatic markers, associations were seen between most markers and the CA score, fibrosis score, and inflammation score.

### Associations between histopathological findings

For the histopathological features applied, the cytoplasmic alteration score was associated with all other features except the PAS-score (Table 5). In fact, the PAS-score showed no association to any of the other histopathological findings. Scores for both fibrosis and inflammatory foci also had a strong association to the quantification of fibrosis (PSR-VIS) (Table 5).

**Table 5:**
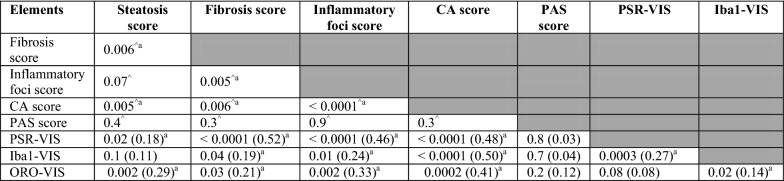
Associations between the categorical and the parametric histopathological findings

PSR-VIS, Iba1-VIS and ORO-VIS as outcome were log-transformed

*CA* cytoplasmic alterations, *Iba1*-*VIS* quantification of macrophages on Iba1 immunohistochemical stain, *ORO*-*VIS* quantification of lipid on oil red O stain, *PAS* positive periodic acid Schiff stain for glycogen, *PSR*-*VIS* quantification of collagen on picro-sirius red stain

Data presented as *p*-value (R^2^)

^^^*p*-value from Fisher’ exact test

^a^Significant result

### Associations between biochemical analyses of liver tissue content and histopathological findings

As expected, tissue triglyceride content showed a strong association to the quantification of lipid (ORO-VIS) and glycogen content was strongly associated to the PAS score (Table 6), even though the PAS ± diastasis staining technique can be challenging in terms of accuracy. In addition, a significant association was found between liver tissue content of cholesterol and most histopathological findings, especially CA score.

**Table 6:** Associations between the biochemical analyses of liver tissue content and the histopathological findings

Variable	Triglycerides^^^	Cholesterol^^^	Glycogen^^^
Parametric findings			
ORO-VIS (lipid)	< 0.0001 (0.59)^a^	0.0004 (0.27)^a^	0.0001 (0.32)^a^
PSR-VIS (collagen)	0.4 (0.04)	< 0.0001 (0.34)^a^	0.6 (0.01)
Iba1-VIS (inflammation)	0.3 (0.03)	< 0.0001 (0.39)^a^	0.9 (0.001)
Categorical scores			
Steatosis	0.006 (0.17)^a^	0.004 (0.24)^a^	0.1 (0.09)
Fibrosis	0.4 (0.07)	0.006 (0.27)^a^	0.6 (0.05)
Inflammation	0.3 (0.1)	0.0002 (0.39)^a^	0.9 (0.01)
Cytoplasmic alterations	0.98 (0.00)	< 0.0001 (0.73)^a^	0.2 (0.11)
PAS positive	0.005 (0.29)^a^	0.4 (0.07)	< 0.0001 (0.75)^a^

*Iba1*-*VIS* quantification of macrophages on Iba1 immunohistochemical stain, *ORO*-*VIS* quantification of lipid on oil red O stain, *PAS positive* periodic acid Schiff stain for glycogen, *PSR*-*VIS* quantification of collagen on picro-sirius red stain

Data presented as *p*-value (R^2^)

^^^Transformed

^a^Significant result

### Intra-assay and intra-observer variations

Lastly the intra-assay coefficients of variance (CV), for all three biochemical tissue content analyses were below 5%. For the histopathological assessment of fibrosis, inflammatory foci, cytoplasmic alterations and PAS positive cells the assigned score differed no more than one when performed by the same observer. No intra-observer difference was found for steatosis score.

## Discussion

This study demonstrated that Göttingen Minipigs fed a high fat diet with fructose and cholesterol became obese and developed hepatomegaly with hepatic fibrosis, inflammation and cytoplasmic alterations when compared to animals fed a normal diet. However, only few animals developed marked steatosis, and hepatocellular hydropic degeneration (ballooning) and Mallory–Denk bodies were not observed in any animal. Streptozotocin-induced diabetes did not exacerbate the changes in circulating biomarkers or hepatic histopathology, compared to non-diabetic animals fed a similar diet. The group changed to standard diet for 6 months had no hepatic changes, except from excess collagen deposition.

The large number of animals, the more than 1-year study period, and the comprehensive investigation of several parameters in the present study strengthen the characterization of the hepatic changes in obese Göttingen Minipigs with and without diabetes.

As expected, the FFC and FFC_DIA_ groups developed obesity with higher BW, BF% and dyslipidemia (defined as increased plasma TG and TC) compared to the two groups fed standard diet. The FFC_DIA_ group had significantly higher levels of fasting GLU and FRA confirming hyperglycemia. Plasma ALP and GLDH were increased, whereas an increase in circulating concentrations of ALT and AST was not found, in fact plasma ALT was lower in FFC pigs compared to SD and FFC/SD. Studies of Ossabaw minipigs on the same diet also found no difference in ALT compared to control animals, whereas AST was increased. However, in pigs, GLHD is considered a more reliable marker of acute liver damage, as compared to ALT, which is non-specific in pigs [26]. Also, in human patients with NAFLD, ALT values have not been found to correlate with the degree of histopathological changes [27]. Table 7 provides an overview of relevant NAFLD/NASH characteristics including selected circulating biomarkers from human and pig studies.

**Table 7:** Comparison of selected elements of porcine models of NAFLD/NASH and human NAFLD/NASH

Characteristics	Humans adult type [28]	Humans pediatric type [40]	Göttingen Minipigs (present study)	Ossabaw miniature swine [12]	Ossabaw miniature swine [10]	Bama minipigs [13]	Microminipigs [11]
Steatosis, macro	Yes	Yes	Minimal	3 out of 7	No	No	–
Steatosis, micro	No	No	No	Yes	–	Yes^a^	Yes
Fibrosis, perisinusoidal	Yes/No	No	Yes	Yes	Yes	–	No
Fibrosis, portal	No	Yes	Yes	Yes	Yes	Yes	No
Inflammation, lobular	Yes	No	Yes	4 out of 7	No	Yes	Yes
Inflammation, portal	No	Yes	Yes	No	–	Yes	–
Hepatocellular ballooning	Yes	No	No	Yes^b^	Yes^b^	No	Yes^b^
↑ triglycerides liver content	–	–	No	Yes	No	–	Yes
↑ cholesterol liver content	–	–	Yes	–	Yes	–	Yes^c^
↑ ALT	Yes/No	Yes/No	No	No	No	Yes	No
↑ AST	–	Yes/No	No	Yes	Yes	Yes	Yes
Hypercholesterolemia	–	–	Yes	Yes	Yes	Yes	Yes
Hypertriglyceridemia	Yes	Yes	Yes	Yes	Yes^1^	Yes	No

Selected histopathological elements, biochemical analyses of liver tissue content of lipids and circulating biomarkers from human and different pig studies. The study of Li et al. [43] is not included as relevant information for comparison is missing

*ALT* Alanine transaminase, *AST* Aspartate transaminase, – Not mentioned

^a^Only present in Sudan III staining

^b^Not typical human-like hydropic degeneration (ballooning)

^c^Given as cholesterol esters

^1^Not significantly different from control group

The LW was highly increased in both FFC and FFC_DIA_ groups compared to SD, and despite elevated liver content of cholesterol in both groups, unexpectedly no statistically significant differences were found for triglycerides liver content among the four groups.

Furthermore, the degree of histopathological alterations was less than expected, especially regarding steatosis, despite the presence of metabolic disturbances indicated by the changes in circulating markers. Few animals displayed more than 5% macrovesicular steatosis which is a criterion for the diagnosis of NAFLD in humans [28, 29]. Rodent models on a high fat diet usually report abundant macro- and microvesicular steatosis [30, 31], but previous studies in Ossabaw minipigs on the same diet (5B4L) also reported lack of macrovesicular steatosis, despite extensive liver injury [10, 12]. Others have reported extensive microvesicular steatosis with little or no presence of macrovesicular steatosis in pigs on high fat plus high cholesterol or high sucrose diets [11, 13]. Microvesicular steatosis was rarely seen in our pigs, and the low level of both types of steatosis is consistent both with the biochemical analysis and the quantification of lipid on ORO stained slides. This modest accumulation of lipids in the liver could reflect the fact that the liver in pigs, in contrast to humans and rodents, is not the primary site of de novo lipogenesis [32]. Also, the overnight fasting period before termination might have had some influence on the lipid content in the liver. However, so far it is unclear what is responsible for the significantly increased LW in FFC and FFC_DIA_ groups and it needs to be clarified if hepatocyte hypertrophy or hyperplasia is the reason for the present hepatomegaly.

Hepatocellular ballooning, another criterion for the diagnosis of human NASH, was not seen in our study, but extensive cytoplasmic alterations in hepatocytes were present in FFC and FFC_DIA_ groups. It was speculated that these alterations could be caused by an accumulation of glycogen. Glycogenic hepatopathy is a condition with massive cytoplasmic deposition of glycogen in hepatocytes leading to hepatomegaly, and is mostly seen in patients with poorly controlled type 1-diabetes. The diagnosis is confirmed with liver biopsies and staining for glycogen with PAS [33]. In this study, staining for glycogen with PAS however did not show correlation with CA in either FFC_DIA_ or FFC, and the FFC_DIA_ group also showed decreased glycogen content in their liver tissue in comparison to the FFC group. Instead a strong association between CA and cholesterol content in the liver persisted, and perhaps CA could reflect a functional adaptation to the increased cholesterol load. In humans, it has recently been suggested that dietary cholesterol activates the hepatic stellate cells thereby promoting fibrosis especially if hepatocyte uptake or biliary excretion of cholesterol is inhibited [34].

Mallory–Denk bodies is a characteristic feature of NASH in humans, but can be difficult to identify and often additional IHC staining has to be performed, e.g., using ubiquitin or cytokeratin 8/18 antibody. Lackner et al. found that CK 8/18 was diminished or absent in ballooned hepatocytes compared to normal hepatocytes. They also found that Mallory–Denk bodies were not always present [25]. Others suggest double immunohistochemical staining of CK8/18 and ubiquitin for the optimal detection of hepatocellular injury in human liver [35]. In some mouse models of NASH, Mallory–Denk bodies have been reported morphologically [30, 36, 37], but until now no previous studies with pigs [10–12] or other rodent species [31, 38] have identified Mallory–Denk bodies along with hepatocellular ballooning. Immunohistochemical staining of CK8/18 or ubiquitin in liver tissue of porcine NAFLD/NASH models has not been reported. However, in a mouse model of NAFLD/NASH staining for CK8/18 has been used [31] and found less or absent staining in hepatocytes with ballooning.

The predominantly periportal fibrosis seen in our minipigs is coherent with findings in Ossabaw minipigs [12]. This is in contrast to humans where perisinusoidal fibrosis around the central vein dominates and periportal fibrosis occurs only in later disease stages. A possible explanation for this peripheral deposition of collagen could be the localization of the porcine hepatic stellate cells. These cells are responsible for collagen production when activated and heterogeneity of stellate cells in the porcine liver was described by Wake et al. [39] who found that desmin positive stellate cells were most abundant in the peripheral regions of the classical lobules. Interestingly, two subtypes of NAFLD have been described in pediatric patients; type 1 resembles ‘adult’ NAFLD whereas type 2 is characterized by steatosis, portal inflammation and portal fibrosis [40] and is known to be the most prevalent type in children. The mechanism behind this pediatric portal fibrosis is unknown but perhaps a common pathway for portal fibrosis in children and pigs exist. The hepatic inflammation seen in FFC and FFC_DIA_ groups was not a reflection of a systemic inflammatory state as the circulating inflammatory markers CRP and ALB showed no difference between the four groups.

Interestingly, the FFC/SD group also differed from SD in terms of fibrosis, despite being fed the same standard diet for the last 6 months. This indicates that fibrosis developed in the first 7 months of high fat/high cholesterol-feeding and either did not progress further during the 6 months of healthy dieting, or maybe even regressed.

Diabetes did not exacerbate the hepatic histopathology, although the FFC_DIA_ pigs had substantial dyslipidemia with elevated levels of TC and TG and a liver tissue cholesterol content exceeding that of the FFC group. Previous unpublished studies indicated that 2% cholesterol was needed in order to achieve the desired elevated level of circulating total cholesterol in normal minipigs, whereas diabetic minipigs reached the same level on a 1% cholesterol diet, which was also the case here. Six diabetic pigs were terminated prematurely nearly reducing the group by half, which could have led to lack of statistical power when comparing the FFC_DIA_ group with the FFC group.

Our diabetic animals had a type 1-like diabetes phenotype and were treated with long acting insulin analogue to keep blood glucose around 15 mM. A lower incidence of NAFLD has been found in patients with type 1 diabetes compared to patients with type 2 diabetes, and it is suggested that insulin treatment’s inhibiting effect on lipolysis is responsible for the decreased level of free fatty acids accumulating in the liver. Insulin resistance induced by excessive caloric intake is also known to play a role in hepatic lipid accumulation and the development of NAFLD. Only FFC_DIA_ had elevated fasting blood glucose, but insulin resistance can be present in muscle and liver without hyperglycemia in human patients with NAFLD [41]. Although insulin resistance was not directly assessed in the current study, K_G_, AUC_insulin_ and HOMA-IR values indicated that FFC and likely FFC_DIA_ animals were insulin resistant on a whole-body level, but since no profound steatosis was present, hepatic insulin resistance is less likely to be a major factor in this model.

A limitation to this study was, that 11 animals ended the study prematurely and necropsy reports mentioned enlarged pale livers in all FFC (n = 2) and FFC_DIA_ (n = 6) pigs, but no further analyses were performed. It could be speculated that these excluded animals especially from the FFC_DIA_ group may have exhibited more pronounced hepatic changes thus biasing our results as only animals with less pronounced changes were able to complete the full study period. Another limitation is that no liver biopsies were taken at different time points during the study, especially before the FFC/SD group was changed to standard diet, making it difficult to conclude if the differences between FFC and FFC/SD were due to lack of progression or regression of marked changes already present at the intervention time point.

Overall, this diet-induced obese Göttingen Minipig model with or without diabetes poses some challenges in terms of translatability with human NASH, because some of the cardinal characteristics (abundant steatosis, hepatocellular ballooning and Mallory–Denk bodies, and zone 3 fibrosis) were missing. However, to explain some of the rather unexpected elements of this model, further studies are needed. Additional staining with e.g. CK8/18, ubiquitin, p62 or sonic hedgehog markers could be performed in order to validate if Mallory–Denk bodies (or their precursors) were present in this minipig model. Moreover, evaluation of serum CK8/18 may elucidate if depletion at tissue level could give as consequence an increase in the circulation of keratin fragments that are major components of Mallory–Denk bodies [42]. By elucidating the pathological pathways perhaps combined with gene expression and electron microscopy, the mechanisms leading to CA in hepatocytes and the portal/periportal fibrosis could be further clarified.

## Conclusion

In conclusion, a diet-induced obese Göttingen Minipig model with and without diabetes has been evaluated as a model of human NAFLD including NASH. Even though fibrosis, inflammation and cytoplasmic alterations occurred, cardinal histopathological characteristics such as hepatocellular ballooning and abundant steatosis were lacking despite the presence of overt obesity and dyslipidaemia. Furthermore, diabetes did not seem to exacerbate the hepatic changes developed by the high fat/fructose/cholesterol diet alone. The limited presence of key human-relevant pathological hepatic findings and variation in the model, limits its use in preclinical research without further optimisation.

## Abbreviations

ALB: albumin
ALP: alkaline phosphatase
ALT: alanine transaminase
AST: aspartate transaminase
AUC_insulin_: area under the curve for insulin response
BF%: total body fat percentage
BW: body weight
CA: cytoplasmic alterations
CRP: C-reactive protein
DEXA: dual-energy x-ray absorptiometry
DL: lobus hepatis dexter lateralis
DM: lobus hepatis dexter medialis
FFC: high fat/high cholesterol diet group
FFC_DIA_: diabetic group
FFC/SD: diet normalization group
FRA: fructosamine
GLDH: glutamate dehydrogenase
GLU: glucose
HE: hematoxylin and eosin stain
HOMA-IR: homeostasis model assessment of insulin resistance
Iba1: ionized calcium binding adapter molecule 1
Iba1-VIS: quantification of Iba1 stained inflammation
IHC: immunohistochemistry
LW: liver weight
K_G_: intravenous glucose tolerance index
NAFLD: non-alcoholic fatty live disease
NASH: non-alcoholic steatohepatitis
OCT: optimum cutting temperature compound
ORO: oil red O stain
ORO-VIS: quantification of ORO stained lipid
PAS: periodic acid-Schiff stain
PSR: picro-sirius red stain
PSR-VIS: quantification of PSR stained collagen
SD: lean control group fed standard diet
SL: lobus hepatis sinister lateralis
SM: lobus hepatis sinister medialis
TC: total cholesterol
TG: triglycerides
TMA: tissue micro arrays
